# Editorial: Cold Pressed Oils: A Green Source of Specialty Oils, volume II

**DOI:** 10.3389/fnut.2023.1224878

**Published:** 2023-06-09

**Authors:** Alessandra Durazzo, Mohamed Fawzy Ramadan, Vita Di Stefano, Massimo Lucarini

**Affiliations:** ^1^CREA-Research Centre for Food and Nutrition, Rome, Italy; ^2^Department of Clinical Nutrition, Faculty of Applied Medical Sciences, Umm Al-Qura University, Makkah, Saudi Arabia; ^3^Department of Biological, Chemical and Pharmaceutical Science and Technology (STEBICEF), University of Palermo, Palermo, Italy

**Keywords:** specialty oils, cold-pressed oils, cold technologies, nutrients, bioactive components, adulteration, food composition databases

## Introduction

In recent years, great attention has been given to new sources searching for vegetable oil as an alternative to conventional products, which is linked to the perception of these oils as natural, nutritional, and safe food products with better nutritive/healthy properties. Cold-pressed oils are promoted as specialty oils, and several cold-pressed oils are currently available on the international market.

This Research Topic has explored recent advances in cold-pressed oils by increasing information and knowledge on their composition, physicochemical characteristics, organoleptic attributes, food qualities and nutritional characteristics, oxidative stability, functional and health-promoting traits, and uses and applications.

Owing to the excellent nutritional profile of cold-pressed oils, recently, there has been an increase in interest. Owing to consumer demand for safe and natural culinary items, the cold pressing technique is interestingly replacing traditional extraction methods. At the industrial level, the cold pressing technology has benefits such as lower energy consumption and cheaper investment costs. When compared to solvent extraction, it has a reduced environmental impact and exhibits greater versatility because it is quick and simple to process a variety of different types of seeds. Cold-pressed oils are preferred over refined edible oils because they have higher levels of bioactive compounds such carotenoids, sterols, and phenolics. The presence of more phenolics and tocols in cold-pressed oils may increase their oxidative stability during storage (1).

In the collection of papers under the Research Topic: “*Cold pressed oils: a green source of specialty oils—volume 2*”, four papers have been published.

Seidita et al. reviewed the clinical impact of an extra virgin olive oil-enriched Mediterranean diet on metabolic syndrome by highlighting future prospects of its nutraceutical uses. However, Oboulbiga et al. reviewed the nutritional value, antioxidant properties, health benefits, and nutrition of sesame seed oil to provide collective information on nutritional and medical interest.

It is worth mentioning the research of Fratianni et al., in which the potential therapeutic benefits of unconventional oils were explored, with a focus on the assessment of the potential *in vitro* biological properties of some Rubiaceae, Cucurbitaceae, and Brassicaceae seed oils.

The research of Yu et al. addressed the kinetic modeling of the sesamin conversion into asarinin in the presence of citric acid loading on Hβ.

## Author contributions

All authors listed have made a substantial, direct, and intellectual contribution to the work and approved it for publication.
